# The role of single nucleotide polymorphisms within genes for oxytocin and vasopressin receptors in the presentation and severity of autistic traits

**DOI:** 10.1192/j.eurpsy.2023.288

**Published:** 2023-07-19

**Authors:** K. M. Wilczyński, A. Auguściak-Duma, A. Stasik, L. Cichoń, A. Sieroń, M. Janas-Kozik

**Affiliations:** 1 Department of Psychiatry and Psychotherapy of Developmental Age, Medical University of Silesia, Katowice; 2 John Paul II Paediatric Center in Sosnowiec, Sosnowiec; 3 Department of Molecular Biology, Medical University of Silesia, Katowice, Poland

## Abstract

**Introduction:**

Autism spectrum disorder is a heterogeneous group of disorders that affects virtually every population, regardless of their ethnic or socioeconomic origin. The pathogenesis of ASD is probably multifactorial, based on interactions between genetic and environmental factors. Their key elements are disorders in the field of social communication, establishing and maintaining relationships and the so-called stereotypical and repetitive patterns of interests and activities. However, of the above- mentioned symptoms, the most important are communication disorders, which are the basis for many of the functional difficulties observed in these patients.

**Objectives:**

The aim of the presented study was to analyze the clinical picture of social cognition deficits in males with autism spectrum disorders, and to link its elements with the frequency of alleles of selected polymorphisms within the OXTR and AVPR1A genes.

**Methods:**

The study included 132 people, 77.5% of whom were male (n = 100). 113 participants (85.6%) were diagnosed with autism spectrum disorders confirmed by the ADOS-2 test conducted by a certified diagnostician. In this group, men constituted 76.1% of the population (n = 77). The remaining 28 people did not have a diagnosis of autism spectrum disorders, and in the ADOS-2 study they obtained the result below the cut-off level. The mean age in the whole group was 14.4 years (95% CI: 13.92-14.93).

**Results:**

A higher frequency of the rs53576 A allele and the rs10877969 C allele could be observed than expected on the basis of the European / world population. In the case of the rs7294536 and rs2254298 polymorphisms, no differences in the distribution of alleles in relation to the expected values were observed. In the network analysis reference allele (T) of SNPs rs10877969 was linked to the higher outcome of the “social affect” domain of ADOS-2 and through it influenced ADOS-2 outcome. All other SNPs did not significantly affect neither domain of ADOS-2. Reference allele (A) of rs53576 was linked with higher odds ratio of clinical diagnosis of ASD in logistic regression. Similarly the rs10877969 polymorphism within the AVPR1a gene significantly shaped the risk of autism spectrum disorders, while in the combined analysis with rs7294536 within the haplotype, the observed effect was significantly stronger.

**Conclusions:**

The studied polymorphisms may constitute an element of larger haplotypes which, depending on the number of mutated alleles, may determine the severity of autism spectrum traits, from the neurotypical population, through people with a broad autism phenotype, to people diagnosed with ASD. Further research is required on the potential clinical application of genotype analysis of the studied polymorphisms and on the exact mechanism of their impact on the risk of ASD and the development of social cognition disorders.

**Disclosure of Interest:**

None Declared

